# The emergence of commercial genomics: analysis of the rise of a biotechnology subsector during the Human Genome Project, 1990 to 2004

**DOI:** 10.1186/gm487

**Published:** 2013-09-20

**Authors:** Ilse R Wiechers, Noah C Perin, Robert Cook-Deegan

**Affiliations:** 1 Center for Public Genomics, Institute for Genome Sciences & Policy, Duke University, Box 90141, Durham, NC 27708, USA; 2 Sanford School of Public Policy, Duke University, Box 90239, Durham, NC 27708, USA; 3 Robert Wood Johnson Foundation Clinical Scholars Program, Yale School of Medicine and US Department of Veterans Affairs, 333 Cedar Street, PO Box 208088, New Haven, CT 06520-8088, USA

## Abstract

**Background:**

Development of the commercial genomics sector within the biotechnology industry relied heavily on the scientific commons, public funding, and technology transfer between academic and industrial research. This study tracks financial and intellectual property data on genomics firms from 1990 through 2004, thus following these firms as they emerged in the era of the Human Genome Project and through the 2000 to 2001 market bubble.

**Methods:**

A database was created based on an early survey of genomics firms, which was expanded using three web-based biotechnology services, scientific journals, and biotechnology trade and technical publications. Financial data for publicly traded firms was collected through the use of four databases specializing in firm financials. Patent searches were conducted using firm names in the US Patent and Trademark Office website search engine and the DNA Patent Database.

**Results:**

A biotechnology subsector of genomics firms emerged in parallel to the publicly funded Human Genome Project. Trends among top firms show that hiring, capital improvement, and research and development expenditures continued to grow after a 2000 to 2001 bubble. The majority of firms are small businesses with great diversity in type of research and development, products, and services provided. Over half the public firms holding patents have the majority of their intellectual property portfolio in DNA-based patents.

**Conclusions:**

These data allow estimates of investment, research and development expenditures, and jobs that paralleled the rise of genomics as a sector within biotechnology between 1990 and 2004.

## Background

A cluster of companies that employed genomic technology emerged in parallel to the publicly funded Human Genome Project between 1990 and 2004 [1,2]. The business plans, technologies, size and financial health of these firms differed widely, but they shared a common reliance on methods and technologies associated with the then-new field of genomics: DNA sequencing, DNA manipulation on the chromosome or whole-genome scale, and bioinformatics. These firms drew heavily on the scientific commons, public funding, availability of startup capital, and two-way technology transfer between academic and industrial research.

As originally conceived, the Human Genome Project was a public works project - to construct maps and derive a reference sequence for the human genome and other genomes. Maps and reference sequences were primarily conceived as scientific tools, but they have obvious commercial implications. New genomics firms began to form in the early 1990s, five years after the Human Genome Project was first conceived in 1985. The firms built on three decades of molecular biology and human genetics research to develop a commercial genomics sector within biotechnology. The information tools - maps, sequences, and algorithms - generally (with some exceptions) resided in the public domain through scientific publications in open literature and public databases. The Human Genome Project also relied upon automated DNA sequencing and sample-handling robotics, products of private sector research and development (R&D). Academic research institutions were the first market for instruments, reagents, software and other projects of many genomics firms. Knowledge and technology moved from academe to industry and also from industry into academic research. In the late 1980s, instrumentation for DNA sequencing, mapping, and polymerase chain reaction became a growth sector. The promise of applications in developing pharmaceutical products, diagnostics, and biologics, as well as agricultural uses, led to adoption of genomics by established firms and creation of new firms that extensively used the nascent technologies.

Most of the private genomics R&D investment began in 1992 and 1993 [3], initially among United States (US) firms or foreign firms investing in US genomics R&D. The first wave of such genomics firms formed around the idea of mapping or sequencing the human genome, finding genes associated with known genetic characters, or shifted their focus from other pursuits to those ends.

Public and nonprofit funding for biomedical R&D grew steadily through the 1980s; privately funded R&D, however, grew even faster, surpassing total public and nonprofit funding by the early 1990s [4]. In genomics, the rise of private R&D was even more sudden and pronounced. From very little investment in private genomics at the beginning of the 1990s as the Human Genome Project began, a survey of genomics research funding for the World Health Organization found that private firm expenditures were almost twice the public and nonprofit funding for genomics R&D by 2000 [5].

**Figure 1:**
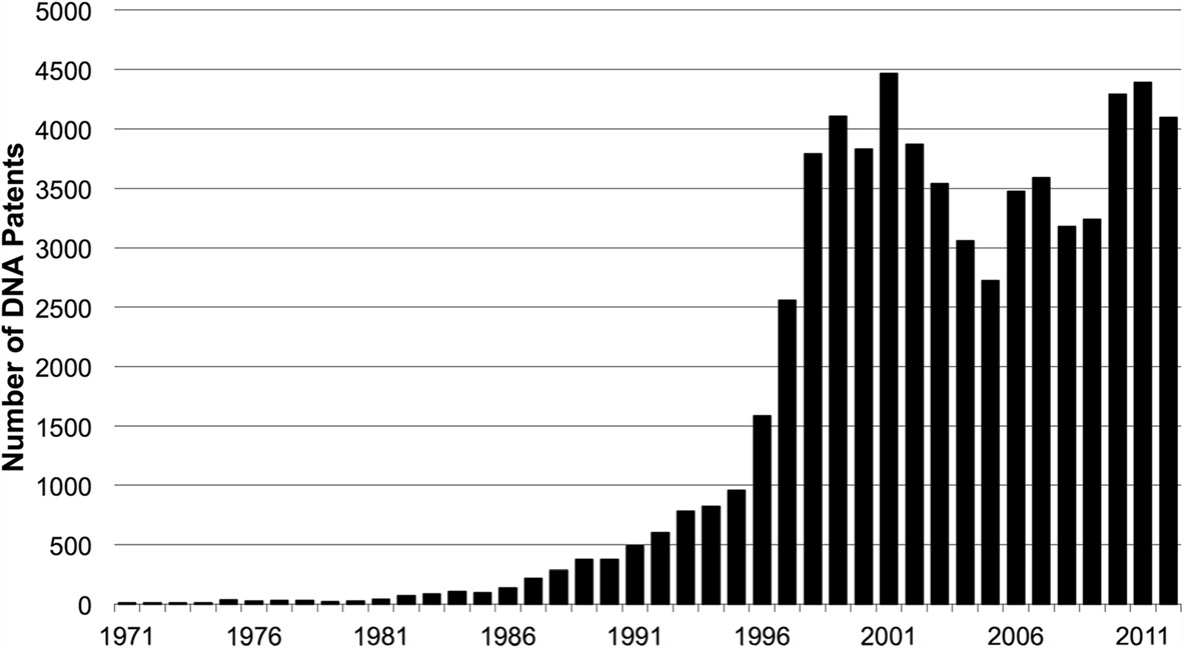
**Number of items loaded into DNA Patent Database, by year through 2013.**[8] Creative Commons ‘free use with attribution’ license, with the attribution to Genomics Policy Resource.

One notable historical feature of genomics is that it grew out of publicly funded science at a time when patent rights were being expanded and strengthened through a combination of changes in legislation, court decisions, and patent office policies. The Human Genome Project was conceived just a few short years after the 1980 passage of the Bayh-Dole Act (Public Law 96–517), which clarified grantees’ and contractors’ rights to seek patents on federally funded research results [6]. Academic institutions expanded their patenting following Bayh-Dole [7], and genomics is one of the areas where the effect was pronounced. The number of DNA patents - that is, US patents that refer to a DNA-specific term in their claims - surged dramatically during the 1990s (see Figure 1).

Technology transfer greatly stimulated the nascent genomics sector; many genomic technologies relating to DNA sequencing and genetic mapping spent a period of gestation in academic R&D. Yet many of the instruments (for example, automated DNA sequencing machines), and some of the most important techniques (such as polymerase chain reaction), were developed in private firms through industrial R&D. The market for genomic technologies is thus a complex hybrid of public and private R&D laboratories, with many technologies starting in private R&D with the goal of development, at least in part, for the academic research market. This history gives genomics firms a distinctive business model; one that involves both direct funding through government grants and contracts and indirect funding through sales of products and services to federally funded or nonprofit laboratories. This is by no means unique, but the degree to which private instrumentation and biotechnology companies developed was remarkable and happened in a short period, less than a decade.

### What is genomics?

The definition of ‘genomics’ derives from Tom Roderick, as first cited in print by McKusick and Ruddle in the inaugural editorial for the new journal *Genomics* in 1987 [9]. At that time, genomics distinguished large-scale mapping and sequencing efforts from molecular studies of one or a few genes [10]. The term genomics gained popularity and came to describe a rapidly growing field of molecular biology, applying to large-scale, rapid DNA analysis, and intensive use of instruments and new technologies. Lederberg and McCray noted that by 2001, ‘genomics’ had acquired a broader meaning, referring to any study that involved the analysis of DNA sequence and even to the study of how genes affect biological mechanism and phenotype [11]. In current parlance, it generally means studies that generate enormous rates of data flow and require extensive computation centered on DNA structure and function, particularly DNA sequencing or metrics of DNA variation.

This work aims to describe commercial genomics as it emerged in parallel to the publicly funded Human Genome Project. We present here descriptive financial and intellectual property data on genomics firms between 1990 and 2004. This allows us to track the sector through the market blip in March 2000, when Prime Minister Tony Blair and President Bill Clinton made a public announcement about DNA sequence patents that led to a dramatic but transient drop in stock valuations, as well as the bubble in late 2000 and into early 2001. Our data provide a glimpse of some underlying trends in the financial inputs and scientific outputs of genomics as it emerged as a subsector within biotechnology (see Major Findings).

### Major findings

•Genomics firms engaged in more than 20 distinct types of business activity.

•Hiring, capital improvement, and R&D expenditures continued to grow despite a tremendous loss of market capitalization in 2000 through to 2001.

•The ability to identify patents referencing DNA-specific terms allows a measure of ‘genomics intensity’ of R&D. The genomics firms identified, with a few exceptions, were highly concentrated in genomics.

•Genomics firms grew to employ at least 28,000 people by 2004.

•Most genomics firms were small businesses.

## Methods

This description of an emerging sector was enabled by two data sources: a database of firms and R&D expenditures initiated at Stanford University and continued at Duke University through 2004 (when the company database was discontinued for reasons described below); and the ability to identify patents making claims that specify terms distinctive to DNA or RNA. The data also construct a window through which to view technology transfer, patenting, and academic-industrial-government interactions, using genomics as an important subset of biotechnology.

### Identifying genomics firms

The definition of what is or is not a genomics firm is somewhat amorphous. Similar to how the term biotechnology refers to the practice of a subset of pharmaceutical firms employing molecular biological methods, genomics is an approach, not an industrial sector. One unifying feature among the many companies that became known as genomics firms and were included in our database was that all or a substantial fraction of their business plans hinged on use of large datasets on several or many genes, emerging DNA technologies of sequencing, novel methods of DNA detection, or interpretation of information based on DNA sequence or structure. This is not restricted to human DNA, but also includes microbes, plants, and other organisms. However, it became increasingly difficult to determine exactly what portion of a firm’s business was related to genomics as the technologies ramified into many disparate lines of life sciences and industrial application. R&D allocations by firms on our list range from complete dedication to genomics to only a small, but meaningful, fraction of R&D funds attributed to genomics. With this in mind, our dataset of genomics firms is a best effort estimation of the genomics sector as it emerged, but should not be viewed as an exact valuation of how much genomics R&D was taking place in the commercial sector.

We used the following criteria to include firms in our analysis: analysis of DNA structure a core business; ‘genomics’ listed on website, annual report, or in news stories as part of the business plan; and firm listed as ‘genomics’ by stock analysts or trade press (subject to correction if determined not to meet one of the above criteria). We accepted the definitions of those reporting the figures (including the trade press characterization of private firms). When reporting on firms and funding programs, we visited websites or read publicly available data sources. We excluded firms solely or primarily focused on protein, rather than DNA structure, or those that identified themselves as primarily ‘proteomics’ or some other ‘-omics’ field other than genomics. These distinctions were not entirely consistent, details about the technologies used were not always explicit, and the amount of information publicly available varied widely. Many firm descriptions made it difficult to make judgments. The rule of thumb was to exclude firms unless they (or others writing about them) explicitly referred to genomics, or when the nature of their business seemed similar to other firms already on the list. In cases of doubt, firms were contacted for clarification, and excluded or included according to the taxonomy noted below.

### General firm information

The database of genomics firms began from two sources: a December 1993 survey of early genomics firms done by one of the authors (RCD; contract report available at the National Reference Center for Bioethics Literature, Georgetown University) [3], and the BioWorld Report *2000 Genomics Review*[12]. Our list was then expanded using several principal sources: three web-based biotechnology services (BioSpace.com, Recombinant Capital, and GenomeWeb.com), scientific journals, and biotechnology trade and technical publications. A few firms were also identified by membership in the Biotechnology Industry Organization or brought to our attention by scientists, stock analysts, or other firms on our list. The database of genomics firms was maintained through 2004, the year after the Human Genome Project formally ended with publication of the human reference sequence in April 2003 [13].

To assemble contact information on firms, we visited the websites for each firm (except the few lacking websites), and made phone calls to clarify points of uncertainty. Our monitoring was greatly expedited by use of the following sources: news about genomics firms in BioSpace.com’s daily ‘Breaking News’ service; twice daily GenomeWeb Daily News Bulletins; Genomics Today, a news service of the Pharmaceutical Research and Manufacturers Association; and reading scientific and trade journals.

We made efforts to gather the following information for each firm in the database: current and former names; contact information such as address, phone, fax, website, and executive officers; year founded; firm taxonomy (as described below); and total number of issued US patents and DNA-based patents (see description of search methods below).

Each firm was designated as being either public, private, acquired, subsidiary, nonprofit, dissolved, or lost to follow-up. Firms that had undergone merger were classified under the acquired category. A firm was designated as dissolved only when direct evidence of dissolution of the business was uncovered (for example, press report, direct contact with former management or staff). All other firms that we could not locate (by web search, or former phone or email contact) but for which we did not have direct evidence of dissolution were considered lost to follow-up.

The database of genomics firms was discontinued in 2004. This was partly a choice to end the study with completion of the Human Genome Project, partly because the data-collection effort was substantial and our research project ended, and partly because the term ‘genomics’ became difficult to justify as a coherent, distinctive category as genomic technologies became ubiquitous in the life sciences and in industrial applications. The problem of definitional wobble is apparent even in government funding programs devoted to genomics, although reasonable estimates were possible for nonprofit and government funding streams through 2008 [14].

One of the limitations of our survey is the relative dearth of trade press or other sources for collecting information about firms outside North America and Western Europe. We acknowledge that our coverage is not uniform and that we may have missed a significant number of international companies. Firms in India, China, other parts of Asia, Latin America, and Eastern Europe are very likely under-represented. This bias applies to publicly traded firms, but is true *a fortiori* for privately held firms, which can be very difficult to identify and monitor.

### Business taxonomy

A genomics taxonomy emerged from reviewing descriptions of R&D carried out by the firms that were described by themselves, on websites or in annual reports, or by others in the trade press and news websites as ‘genomics firms.’ The categories emerged from a bootstrapping process of classifying companies, comparing results of classification among the research team, adding terms to accommodate new categories up to a point of ‘saturation’ when few reclassifications were needed; inter-rater reliability was established. Categories in the taxonomy are not mutually exclusive; each firm can be classified under multiple headings.

### Financial data

For publicly traded genomics firms, we gathered the following additional annual financial data: total operating expenses, R&D expenses, number of employees, plant and equipment values, total revenues, net income, and market capitalization. Market capitalization was either gathered directly from financial data sources or was calculated by taking the product of the adjusted closing value of the stock on the day of fiscal year end and the reported number of outstanding shares in the annual financial reports.

Financial data for publicly traded US and international firms were collected primarily through the use of four databases specializing in firm financials: Mergent Online - U.S Company Data [15], Compustat North America [16], Thomson Research - Worldscope [17], and OneSource - Business Browser [18]. The source of these databases’ information is US Securities and Exchange Commission filings, press releases, and analyst reports. In some cases, when companies were not listed in one of these databases, we gathered data directly from firm annual reports. Despite accessing multiple data sources, there remain several firms for which we were unable to locate all financial data points. (Data tables are included in supplemental materials, see Additional file 1) Our aggregate data are thus only a rough proxy for collective activity in private commercial genomics, not comprehensive and fine-grained analyses of particular firms or technologies.

### Patent searches

To obtain the count of total issued US patents, we conducted searches using the US Patent and Trademark Office website search engine [19]. Searches were done looking for the name and former names of each firm as the assignee on patents. Efforts were made to incorporate the patents of acquired and subsidiary firms into the total count of patents for parent firms. We also searched for common misspellings and typos for firm names, when appropriate. Total issued US patent counts were current through 7 February 2006, covering two years beyond the period for which we report company financial data. This two-year period approximates the time of traditional total pendency for patents at the US Patent and Trademark Office [20].

The many distinctive terms for DNA and RNA allow DNA patents to be identified with a relatively high degree of specificity and sensitivity, providing an analytical tool to study genomic innovation. To obtain the count of DNA-based patents, we conducted searches for issued US patents in the DNA Patent Database (DPD) [21]. Established in 1994, the DPD contains patents (and, since 1999, patent applications) with one or more claims explicitly referring to DNA or RNA or terms of art specific to DNA (for example, ‘plasmid’ or ‘nucleotide’), mapping patents to the field of genomics. This patent collection goes well beyond just gene patents (usually referring to DNA molecules encoding proteins) to include methods, instruments, and software. The search algorithm is available online [22]. The individual terms used in the DPD were tested individually for specificity and sensitivity in 1997 and the algorithm modified and re-tested in 2003. Our searches were performed using the 2003 algorithm and utilized techniques similar to those described above for total issued US patents. DPD patent counts cited here are up to date through 11 January 2006, also covering two years beyond the period for which we report company financial data. Comparing DNA patents to total patents yields a ratio of genomics to other patenting activity, a rough indicator of ‘genomics intensity.’

## Results

The final database contained information on 470 genomics firms from 25 different countries, 1990 through 2004. The majority of publicly traded and privately held genomics firms in our database were in the US; 75% and 62%, respectively. Canada, Germany, France, United Kingdom, and Japan rounded out the top six countries (see Table 1).

**Table 1:** Top countries with genomics firms

**Country**	**Public**	**Private**	**Nonprofit**	**Total**
United States	65	130	1	196
Canada	6	17	1	24
Germany	6	15	0	21
France	0	10	0	10
United Kingdom	3	6	0	9
Japan	0	5	0	5

The firms by type in 2004 were: private (211, 45%), public (88, 19%), acquired (90, 19%), subsidiary (27, 6%), dissolved (23, 5%), nonprofit (2, 0.4%), and lost to follow-up (29, 6%). Thus 30% of firms had either dissolved, been acquired, or had become subsidiaries of another larger firm since 1990. The number of publicly traded firms was 88 in 2004, reaching this peak in 2002 and subsequently stabilizing. Consolidation occurred after 2001, as established pharmaceutical and biotechnology firms sought to fill gaps in their research programs or intellectual property holdings. In addition, smaller firms merged. Consolidation in part explained the leveling effect on the overall numbers of publicly traded firms.

Review of the genomics taxonomy revealed the R&D being completed by firms in our database included almost 20 different classifications of research, ranging from agricultural genomics to DNA sequencing to forensics to drug development (see Table 2). The most common category for both public and private firms was ‘drug, biologic and vaccine development;’ accounting for 55% of public firms (for example, Millennium Pharmaceuticals) and 33% of privately held firms (for example, AGY Therapeutics). Approximately one quarter of public firms were in the business of providing ‘instruments for DNA analysis’ (for example, Affymetrix). Another 20% of public firms conducted business as ‘genomics reagents supplier; microarray manufacturer; service provider’ (e.g. Invitrogen) or ‘DNA testing service, clinical or diagnostic screening service, test kit manufacturing’ (e.g. Gen-Probe). Almost 30 percent of private firms were involved in ‘bioinformatics for DNA analysis; data mining’ (for example, DNAStar) and over 20% conducted ‘gene expression analysis; microarray analysis; or analysis of siRNAs and other regulation element’ (for example, Ipsogen).

**Table 2:** Genomics firms taxonomy

**Category**	**Description**
AGRIVET	Agriculture and veterinary genomics
DATABSE	Database creation, subscription, or licensing
DNASEQN	DNA sequencing
DNATEST	DNA testing service, clinical or diagnostic screening
DRUGDEV	Drug, biologic, and vaccine development
GENEFNL	Gene function and functional genomics; characterization of genes and their products
GENEMAP	Gene mapping; linkage, association studies; SNP discovery, use and analysis
GENEPOP	Genetic epidemiology; population studies
GENETFR	Gene transfer and gene therapy; vectors for gene therapy
GENEXPR	Gene expression analysis; microarray analysis; analysis of siRNAs and other regulation element
IDNTFCN	DNA forensics, DNA identification service
INFRMTX	Bioinformatics for DNA analysis; data mining
INSTRMT	Instruments for DNA analysis
LEGLSVC	Legal services; privacy protection
PHRMGEN	Pharmacogenetics or pharmacogenomics
STANDRD	Setting standards, testing service benchmarks
SUPPLYR	Genomics reagents supplier; microarray manufacturer; service provider
TRSTFND	Trust fund or genomics capital source
SYNBIOL	Synthetic biology

### Financial data on publicly traded genomics firms

**Figure 2:**
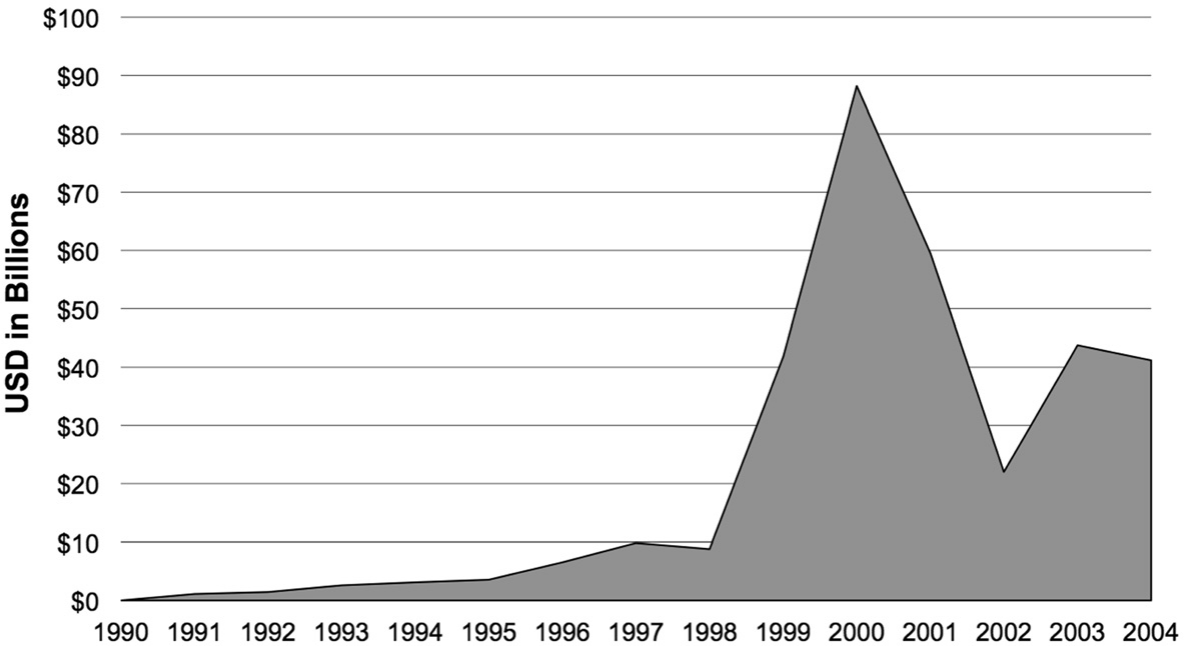
Aggregate market capitalization of all genomics firms.

**Figure 3:**
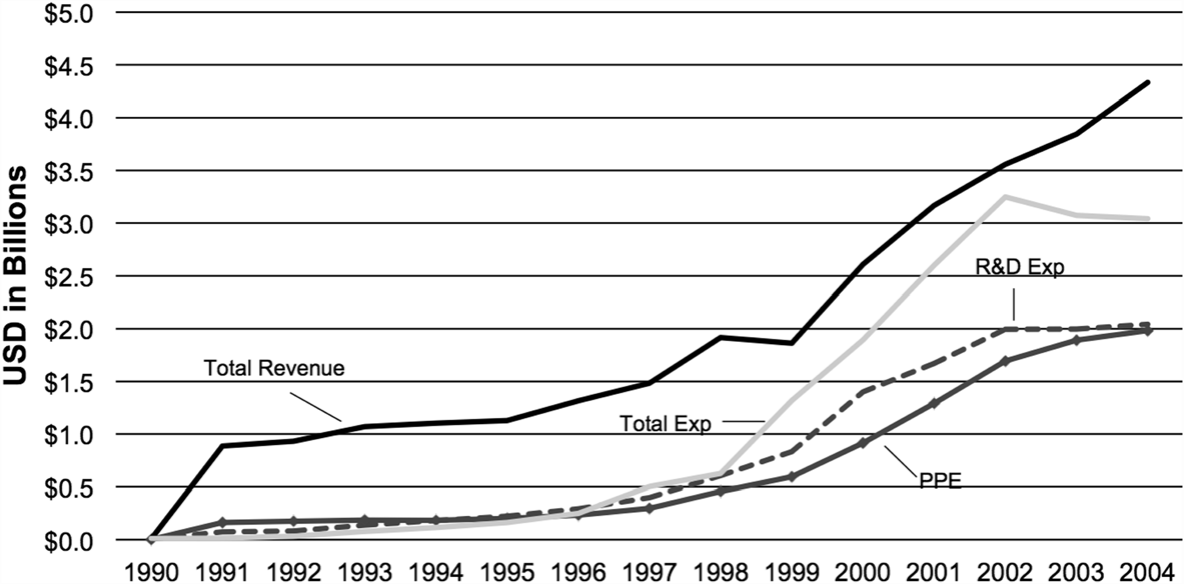
**Financial trends of top 15 public genomics firms.** Top 15 genomics firms by market capitalization are: Applera, Millennium Pharmaceuticals, Invitrogen, OSI Pharmaceuticals, Gen-Probe, Affymetrix, Protein Design Labs, Human Genome Sciences, ZymoGenetics, Abgenix, Incyte, Digene, Exelixis Pharmaceuticals, Lexicon Genetics, and Rigel Pharmaceuticals. PPE, plant, property and equipment; R&D Exp, research and development expenditures; Total Exp, total expenditures.

Total global market capitalization for the publicly traded firms dropped 52% from the 2000 peak value of over $84 billion to the 2004 value of $40 billion (see Figure 2). The top 15 genomics firms, based on market capitalization value in 2004, represented over 70% of the total genomics sector’s value, over $28 billion. These top 15 firms spent a combined $2 billion in R&D, generated $4.3 billion in annual revenues, and had just under $2 billion in plant, property, and equipment in 2004 (see Figure 3). Analysis of the top 15 firms demonstrated broad growth trends, indicating that R&D and capital improvement continued to increase both before and after the 1998 to 2001 bubble, despite the 2000 peak and following decline in market capitalization. These trends in the top 15 firms paralleled the consistent growth in total revenues and R&D expenditures for all publicly traded firms.

**Figure 4:**
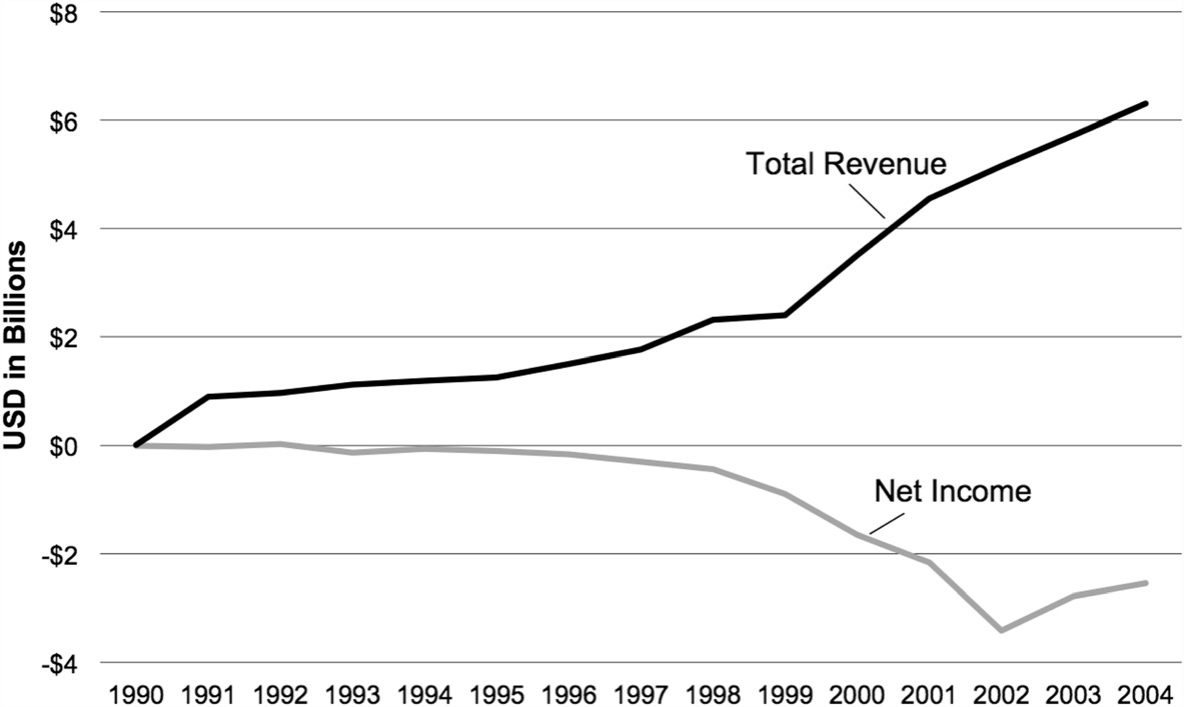
Aggregate net income and total revenue for all genomics firms.

The majority of genomics firms were not profitable by the end of 2004. Even those considered successful and ranking in the top 15 by market capitalization had an aggregate net income in 2004 of negative $1.2 billion. However, beginning in 2003, net income for the sector had begun to trend upwards, that is, there was an aggregate reduction in losses (see Figure 4). Through 2004, total revenues for the genomics sector continued to climb, and in 2004 generated approximately $6.3 billion in revenues, with a combined net income of negative $2.5 billion.

### Employment trends

**Figure 5:**
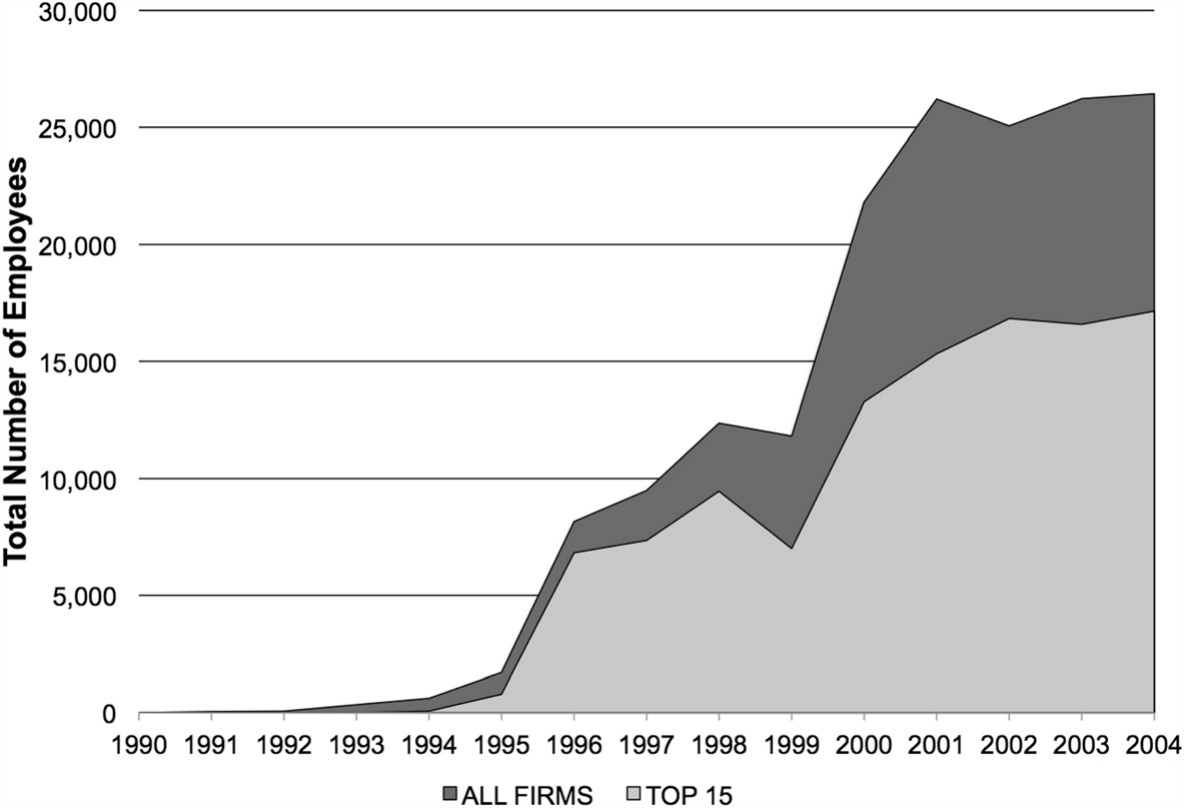
**Employment trends.** Top 15 genomics firms by market capitalization are: Applera, Millennuim Pharmaceuticals, Invitrogen, OSI Pharmaceuticals, Gen-Probe, Affymetrix, Protein Design Labs, Human Genome Sciences, ZymoGenetics, Abgenix, Incyte, Digene, Exelixis Pharmaceuticals, Lexicon Genetics, and Rigel Pharmaceuticals.

Employment trends in the top 15 genomics firms by market capitalization showed that hiring increased both before and after the bubble, reaching its highest point during the study period in 2004 at over 17,000 people (see Figure 5). The trend of the sector as a whole was similar, though there is evidence of a decrease between 2001and 2002, which may partly be explained by data drop out occurring after 2002. Some firms dropped out due to acquisition or dissolution during this time period, but others with missing data were still functioning and reporting financial data during those years. Employment for the sector as a whole was at its highest point during the study period in 2004, with almost 28,000 employees among firms reporting employment data.

Based on the US Small Business Administration Size Standards, matched to the North American Industry Classification System (NAICS) (using size standards for NAICS code 541711 for ‘Research and Development in Biotechnology’), the overwhelming majority (83%) of genomics firms in 2004 were classified as small businesses (employing fewer than 500 people) [23]. In fact, almost one third of genomics firms employed fewer than 100 people.

### Intellectual property outputs

Another measure of output for genomics firms and a potential proxy for productivity is number of patents issued. There were 5,859 US patents owned by active and independent genomics firms in our database. Eighty-nine percent of public firms held patents, with the top 10 firms (by US patent count) holding nearly 60% of the total US patents. There were 3,683 DNA-based US patents owned by active and independent genomics firms. The top 10 firms (by DNA patent count) held 64% of all DNA patents. Among those firms, the percentage of their intellectual property portfolio attributed to DNA patents ranged from 44% to 93%, giving an indication of ‘genomics intensity’.

This level of genomics intensity held true for the sector at large, not just the top 10 firms. Although not all firms held patents, DNA-based patents comprised over half of the patent portfolio among 53% of publicly traded firms that had patents.

The financial, employment, and intellectual property data from the publicly traded genomics firms are available in Additional file 1.

## Discussion

The emergence of a genomics sector of biotechnology was captured from 1990 through 2004, near the beginning of its emergence as the Human Genome Project began, to a year after the human reference sequence was successfully produced. The ability to track DNA-specific patents and an ongoing database of firms maintained by a sequence of students at Stanford and Duke universities enabled tracking at the firm level. The count of firms, and their employment and R&D expenditures, and patent outputs may be of interest to those studying the emergence of the genomics sector, or those who study quantitative aspects of innovation and the emergence of new technologies.

Our definition of a genomics firm includes some firms that did not base their products and services on quintessential genomic technologies (such as high-throughput sequencing or genome-wide analysis) or did not focus on human DNA. Digene, for example, focused on human papillomavirus diagnostics before it was acquired by QIAGEN (after the period of our study), and Myriad focused on *BRCA* genetic testing (of just two genes, not many) for most of the period in our dataset. Some firms were established before the term genomics became broadly used in 1987. We included firms that fit into one or more of the taxonomy categories, presented themselves as genomic in part, or whose R&D included technologies captured by patents in the DPD. Thus, it is important to note that some firms in our database are not genomic in the narrower sense in which it is often used, and not all are human or medically focused; our data should be interpreted accordingly.

The taxonomy of activities carried out in genomics firms captures the breadth of economic sectors and the mix of products and services enabled by genomic technologies, and categorization of firms gives a rough sense of how many firms engaged in those activities. This breadth of products, services, and business models must expand even further among today’s firms in the era of next-generation sequencing. The challenges of big data require creative and unique approaches to not only the science but also its funding [24].

One feature that emerges from the data is the extraordinary growth of market capitalization value of genomics firms for the better part of a decade until a blip in March 2000. The valuations generally recovered and continued to grow until after the June 2000 announcement of a draft human genomic sequence. In the later part of 2000, however, a bubble burst in both genomics and information technology stocks, leading to a five-fold decrement in valuation of the top 15 firms. One interesting finding from our data is that these firms nevertheless continued to increase their R&D expenditures in 2000 and subsequent years, despite the dramatic drop in overall firm valuation. This suggests these firms remained focused on R&D-intensive business strategies, with exit, sale, or profitability dependent on pursuing successful research pathways to products and services, making R&D expenditure important to sustain even in an adverse financial climate. A ‘continue to research through the storm’ strategy appears to have been pursued by most firms.

The aggregate statistics reported here are best interpreted in light of case studies of technologies or in studies of application areas. The picture that emerges by combining aggregate statistics and individual case studies is richer than either method alone. For example, there are numerous genomics firms that show the intricate mutualism between academic and industrial R&D. DNA sequencing was developed in nonprofit research institutions and the prototype instrument for automated sequencing was developed at the California Institute of Technology, but refinement and development of the instrument drew on engineering and manufacturing expertise in the startup firm Applied Biosystems [25]. Polymerase chain reaction was discovered at Cetus in 1983, but found immediate and widespread use in scientific laboratories, and eventually yielded over $2 billion in revenues before the initial patents began to expire [26]. These are just two of many examples of industrial-academic technological interactions that underlie the data captured by R&D expenditures, market capitalization, patenting, and employment figures reported here.

## Conclusions

These data allow estimates of investment, R&D expenditure, and employment that paralleled the rise of genomics as a sector within biotechnology between 1990 and 2004. Financial trends show hiring, capital improvement, and R&D expenditure continued to grow after the 2000 to 2001 market bubble. There was a great diversity in the type of work done by genomics firms, most of which were small businesses with a majority of their intellectual property portfolio in DNA-based patents.

## Competing interests

The authors declare that they have no competing interests.

## Authors’ contributions

IRW oversaw and participated in data collection and data analysis, and drafted the manuscript. NCP participated in data collection, data analysis, and revisions of the manuscript. RC-D conceived of the study, oversaw data collection and data analysis, and helped draft the manuscript. All authors read and approved the final manuscript.

## Authors’ information

IRW was a post-doctoral fellow at the Duke Center for Public Genomics. She has since completed her training in geriatric psychiatry and is currently in the Robert Wood Johnson Foundation Clinical Scholars Program at Yale School of Medicine. NCP was a Masters student in Public Policy at Duke University. Currently he is a Commercialization Officer at PATH, a global health NGO headquartered in Seattle Washington. RC-D is a Research Professor at the Sanford School of Public Policy and the Institute for Genome Sciences & Policy at Duke University.

## Supplementary Material

Additional file 1 Microsoft Excel file containing all of the financial, employment, and intellectual property data from the publicly traded firms in our database. Click here for file
